# Myocardial T_1_-mapping at 3T using saturation-recovery: reference values, precision and comparison with MOLLI

**DOI:** 10.1186/s12968-016-0302-x

**Published:** 2016-11-18

**Authors:** Sebastian Weingärtner, Nadja M. Meßner, Johannes Budjan, Dirk Loßnitzer, Uwe Mattler, Theano Papavassiliu, Frank G. Zöllner, Lothar R. Schad

**Affiliations:** 1 Computer Assisted Clinical Medicine, University Medical Center Mannheim, Medical Faculty Mannheim, Heidelberg University, Theodor-Kutzer-Ufer 1-3, 68167 Mannheim, Germany; 2 Electrical and Computer Engineering, University of Minnesota, Minneapolis, MN USA; 3 Center for Magnetic Resonance Research, University of Minnesota, Minneapolis, MN USA; 4 DZHK (German Centre for Cardiovascular Research) partner site Heidelberg/Mannheim, Mannheim, Germany; 5 Institute of Clinical Radiology and Nuclear Medicine, University Medical Center Mannheim, Medical Faculty Mannheim, Heidelberg University, Mannheim, Germany; 6 1st Department of Medicine Cardiology, University Medical Center Mannheim, Medical Faculty Mannheim, Heidelberg University, Mannheim, Germany

**Keywords:** Saturation-recovery T_1_-mapping, SAPPHIRE, SASHA, MOLLI, 3T, Reference values, Cardiovascular magnetic resonance

## Abstract

**Background:**

Myocardial T_1_-mapping recently emerged as a promising quantitative method for non-invasive tissue characterization in numerous cardiomyopathies. Commonly performed with an inversion-recovery (IR) magnetization preparation at 1.5T, the application at 3T has gained due to increased quantification precision. Alternatively, saturation-recovery (SR) T_1_-mapping has recently been introduced at 1.5T for improved accuracy.

Thus, the purpose of this study is to investigate the robustness and precision of SR T_1_-mapping at 3T and to establish accurate reference values for native T_1_-times and extracellular volume fraction (ECV) of healthy myocardium.

**Methods:**

Balanced Steady-State Free-Precession (bSSFP) Saturation-Pulse Prepared Heart-rate independent Inversion-REcovery (SAPPHIRE) and Saturation-recovery Single-SHot Acquisition (SASHA) T_1_-mapping were compared with the Modified Look-Locker inversion recovery (MOLLI) sequence at 3T. Accuracy and precision were studied in phantom. Native and post-contrast T_1_-times and regional ECV were determined in 20 healthy subjects (10 men, 27 ± 5 years). Subjective image quality, susceptibility artifact rating, in-vivo precision and reproducibility were analyzed.

**Results:**

SR T_1_-mapping showed <4 % deviation from the spin-echo reference in phantom in the range of T_1_ = 100–2300 ms. The average quality and artifact scores of the T_1_-mapping methods were: MOLLI:3.4/3.6, SAPPHIRE:3.1/3.4, SASHA:2.9/3.2; (1: poor - 4: excellent/1: strong - 4: none). SAPPHIRE and SASHA yielded significantly higher T_1_-times (SAPPHIRE: 1578 ± 42 ms, SASHA: 1523 ± 46 ms), in-vivo T_1_-time variation (SAPPHIRE: 60.1 ± 8.7 ms, SASHA: 70.0 ± 9.3 ms) and lower ECV-values (SAPPHIRE: 0.20 ± 0.02, SASHA: 0.21 ± 0.03) compared with MOLLI (T_1_: 1181 ± 47 ms, ECV: 0.26 ± 0.03, Precision: 53.7 ± 8.1 ms). No significant difference was found in the inter-subject variability of T_1_-times or ECV-values (T_1_: *p* = 0.90, ECV: *p* = 0.78), the observer agreement (inter: *p* > 0.19; intra: *p* > 0.09) or consistency (inter: *p* > 0.07; intra: *p* > 0.17) between the three methods.

**Conclusions:**

Saturation-recovery T_1_-mapping at 3T yields higher accuracy, comparable inter-subject, inter- and intra-observer variability and less than 30 % precision-loss compared to MOLLI.

**Electronic supplementary material:**

The online version of this article (doi:10.1186/s12968-016-0302-x) contains supplementary material, which is available to authorized users.

## Background

The recent introduction of rapid parameter mapping into cardiovascular magnetic resonance (CMR) imaging provides the invaluable ability for noninvasive quantitative myocardial tissue characterization. The quantification of the native longitudinal magnetization recovery time as a spatially resolved map (native T_1_-mapping) shows promising prognostic and diagnostic value in various cardiomyopathies [1]. The combination with post-contrast T_1_-time measurements allows for the estimation of the extracellular volume fraction (ECV), which reflects fibrotic remodeling [2], a common endpoint of many pathological cardiac conditions [3].

A number of cardiac T_1_-mapping methods have been proposed, each offering a distinct profile of advantages. The modified Look-Locker inversion recovery (MOLLI) sequence [4] and variations thereof, like the shortened MOLLI (ShMOLLI) [5], are commonly used for myocardial T_1_-mapping. However, confounding factors to the method’s quantification accuracy including heart rate [6], T_2_ relaxation time [7], and magnetization transfer [8] lead to underestimation of the T_1_-time of the healthy myocardium by ~20 % at 1.5T [9, 10].

Alternatively, saturation-recovery (SR) based myocardial T_1_-mapping methods have been proposed [11] and were recently revisited by the SAturation-recovery single-SHot Acquisition (SASHA) sequence [12]. To increase the low dynamic range in SR T_1_-mapping, the hybrid sequence for Saturation Pulse Prepared Heart-rate independent Inversion-REcovery (SAPPHIRE) T_1_-mapping was introduced, using a combination of saturation and inversion pulses for magnetization preparation [6]. While SASHA and SAPPHIRE result in excellent accuracy, the sequences still suffer from reduced precision in assessing T_1_-times compared with MOLLI, as previously shown at 1.5T [9].

The application of inversion-recovery T_1_-mapping at 3T has recently received increasing interest. Multiple studies have shown promising T_1_-map quality and improved quantification precision, due to the increased imaging Signal-to-Noise ratio (SNR) at 3T [5, 13, 14]. Thus, in this work we sought to study the visual quality and precision of SR T_1_-mapping and to establish accurate reference values for native T_1_-times and ECV-values of the healthy myocardium at 3T.

## Methods

All images were acquired on a 3T MRI scanner (Magnetom Skyra; Siemens Healthcare, Erlangen, Germany) with a 30-channel receiver coil array.

### Sequences

T_1_-mapping was performed using the SAPPHIRE and SASHA SR methods and T_1_-times were compared to MOLLI T_1_-mapping. All T_1_-mapping sequences were implemented with a balanced Steady-State Free-Precession image acquisition (bSSFP) and shared the following parameters for phantom and in-vivo imaging: TR/TE/α = 2.6 ms/1.0 ms/35°, in-plane resolution = 1.7 × 1.7 mm^2^, slice-thickness = 6 mm, field-of-view = 440 × 375 mm^2^, bandwidth = 1085Hz/px, number of k-space lines = 139, linear profile ordering, startup-pulses = 5 Kaiser-Bessel, GRAPPA-factor = 2. The 5(3)3 MOLLI scheme was employed for native T_1_-mapping and the 4(1)3(1)2 scheme for post-contrast imaging [15]. For SAPPHIRE and SASHA, 10 images were acquired with the 9 recovery times (inversion or saturation times, respectively) linearly spaced between the minimal (113 ms) and the maximum recovery time, as determined by the duration of the respective R-R interval. Magnetization saturation was achieved using a composite “Water suppression Enhanced through T_1_-effects” (WET) [16] saturation module. An adiabatic full passage tan/tanh pulse [17] was used for magnetization inversion.

### Phantom experiments

Phantom scans were performed to study pulse-efficacy, ex-vivo accuracy and precision of the SR T_1_-mapping methods at 3T. Detailed description of the phantom experiments can be found in the Additional file 1.

### In-vivo experiments

**Fig. 1:**
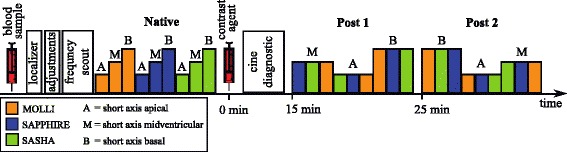
In-vivo imaging protocol: After blood draw, all subjects underwent MR examination of approximately 1 h duration, including T_1_-mapping sessions prior to, 15 and 25 min after GBCA injection. Basic adjustments and frequency scouting were performed before native T_1_-mapping. To minimize the effects of GBCA washout on inter-sequence comparison, measurements of the same slices were grouped. The sequence orders within the group, as well as the slice order were randomized for each subject

20 healthy volunteers (27 ± 5 years, ranging from 20 to 39 years; 10 male: 27 ± 6 years; 10 female: 27 ± 4 years) were recruited for native and post-contrast T_1_-mapping. Figure 1 depicts the schematic of the scan protocol: A blood sample was drawn prior to each examination to measure blood hematocrit for ECV calculation and to exclude impaired renal function before the administration of a gadolinium based contrast agent (GBCA). Imaging was performed before bolus administration of 0.2 mmol/kg gadoterate meglumine (Dotarem; Guerbet, Aulnay-sous-Bois, France), and 15 and 25 min thereafter. T_1_-maps were acquired in three short-axis slices. Based on bSSFP frequency scout images, frequency offsets in the range of ±50 Hz and ±100 Hz were selected for MOLLI and the SR methods, respectively. Different off-resonance frequency shifts were chosen for MOLLI and SR, due to previously reported off-resonance sensitivity for the MOLLI sequence and off-resonance resilience for SR methods [10]. Post-contrast scan order was randomized to mitigate T_1_-trends caused by GBCA washout (Fig. 1).

### Post-processing

Motion correction (MoCo, Advanced Retrospective Technique; Siemens Healthcare, Erlangen, Germany) was applied to co-register the T_1_-weighted image series. T_1_-maps were generated a) from the MoCo image series when the registration algorithm reduced residual motion and b) from the uncorrected image series when MoCo introduced registration distortions, as judged by visual assessment in consensus agreement of two reviewers (SW; 6 years of CMR experience, NMM; 4 years of CMR experience).

MOLLI T_1_-times were obtained using standard post-processing [4] and SR T_1_-maps were generated using a three-parameter fit [18]. Regional ECV-values were calculated segment-wise according to the AHA 16-segment model [19] for both post-contrast time points. Contrast agent concentrations were calculated for the myocardium and the blood-pool, based on the difference in the native and post-contrast relaxation rates (1/T_1_) divided by an assumed relaxivity of 3.5 mmol/L/s [20].

### T_1_-map analysis

Quantitative evaluation of T_1_-times and ECV-values was performed on a per-segment basis. In-vivo precision was defined as the intra-segment variation, measured in terms of standard deviation. Visual T_1_-map quality was evaluated by two readers, which were blinded to the sequence type (JB; >5 years of CMR experience; DL; >11 years of CMR experience). Each slice was scored separately with respect to overall T_1_-map quality (1: poor – 4: excellent) [15] and visual off-resonance artifacts in the T_1_-map (1: strong artifacts – 4: none). The detailed scoring criteria can be found in Additional file 2.

Average T_1_-times and ECV-values were statistically compared on a per-subject basis among the methods using ANOVA, followed by pair-wise paired Student’s t-tests, if significant differences among the methods were detected. The inter-subject variability of the T_1_-times and ECV-values was compared among the methods using a Bartlett-test, and paired F-tests in case of significant results of the former. ECV-values between the two post-contrast time points were compared using a paired Student’s *t*-test for each method. Furthermore, inter- and intra-observer variability was studied for native and post-contrast T_1_- mapping with the three sequences. A total of three ROI sets was independently drawn by two readers for each sequence and time point (Reader 1: UM, 12 years of CMR experience, Reader 2: NMM, 4 years of CMR experience; Reader 1: ROIs A, Reader 2: ROIs B, ROIs C). T_1_-times obtained with different ROI sets were compared on a per subject-basis for inter- (ROIs A vs. ROIs B) and intra-observer (ROIs B vs. ROIs C) analysis. Observer agreement was studied by analyzing the absolute difference between the T_1_-times as proposed in [21]. Observer consistency was assessed using the intraclass correlation coefficient (ICC) based on Winer’s adjustment for anchor points [22]. The T_1_-time variation and the ordinal scaled image ratings were statistically evaluated using Kruskal-Wallis tests with subsequent Mann–Whitney U tests in case of significant difference between the three methods. Differences in the observer agreement were assessed with one-way analysis of variance (ANOVA) of the log-transformed absolute difference [22]. ICCs were statistically compared using two-tailed F-statistics, with Bonferroni correction yielding significance for *p* < 0.017. All other statistical tests were performed at a significance level of *p* < 0.05.

## Results

### Phantom experiments

WET saturation modules resulted in average saturation efficacy >99 % across a broad T_1_-range. The SR methods showed excellent accuracy (<3.9 % deviation). T_1_-time variation was 29 and 50 % lower using MOLLI compared with SAPPHIRE and SASHA, respectively.

### In-vivo experiments

Scanning was successfully completed in all subjects, with no pathological findings. Eight (0.09 %) out of a total of 8640 segments were excluded from further analysis due to imaging artifacts (SAPPHIRE: 4, 0.14 %; SASHA: 4, 0.14 %). Post-contrast results are given for the first time point (~15 min) in the remainder of the study if not explicitly stated otherwise.

**Fig. 2:**
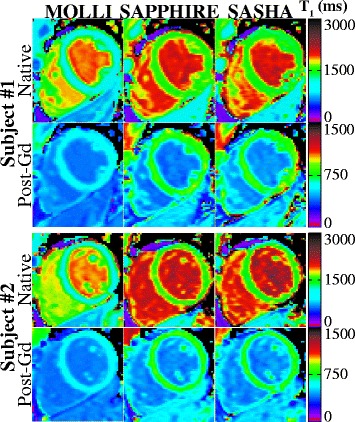
Example T_1_-maps acquired prior to and 25 min after GBCA injection with all three T_1_-mapping sequences in short axis mid-ventricular slices of two healthy subjects. Visually high T_1_-map quality is apparent, with no artifacts and homogenous T_1_-times throughout the myocardium with all three methods. Sharp delineation of the myocardium against the blood-pools is observed for both native T_1_ and T_1_ post-contrast. MOLLI T_1_-maps show systematically lower T_1_-times compared with the saturation-recovery sequences

Figure 2 shows exemplary native and post-contrast T_1_-maps acquired with MOLLI, SAPPHIRE and SASHA in two healthy subjects. All three methods depict a homogeneous myocardium clear of artifacts.

**Fig. 3:**
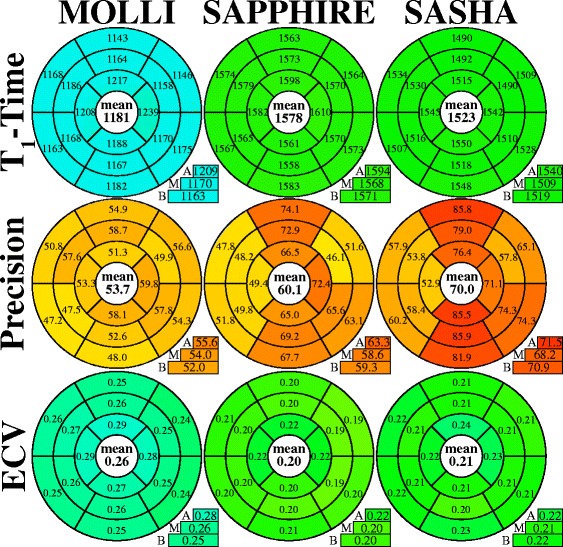
Bullseye plots comparing the native T_1_-times (*top row*), precision (*middle row*) and the ECV-values (*bottom row*) of the three T_1_-mapping sequences averaged over all volunteers. The given ECV-values were calculated from post-contrast T_1_ acquired 15 min after GBCA injection. Segmentation was performed according to the AHA 16-segment model in three short-axis slices (*A* = apical, *M* = mid-ventricular, *B* = basal). The average across all segments is given in the center of the bullseye, the slice averages can be found below. The MOLLI sequence shows lower T_1_, better precision and higher ECV-values compared to the saturation-recovery methods. SAPPHIRE results show similar native T_1_- and ECV-values with slightly better precision compared with SASHA

Native T_1_-time, T_1_-time precision and ECV-values are presented for the 16 AHA segments as bullseye plots in Fig. 3. MOLLI T_1_-times (1181 ± 47 ms) show a 20–29 % underestimation compared with SR T_1_-times (SAPPHIRE: 1578 ± 42 ms, *p* < 0.001; SASHA: 1523 ± 46 ms, *p* < 0.001). SAPPHIRE T_1_-times were slightly higher than SASHA T_1_-times (difference: 3.5 ± 1.9 %, *p* < 0.001). No significant difference was found between the inter-subject variabilities of the three methods (MOLLI: 47 ms, SAPPHIRE: 42 ms, SASHA: 46 ms, *p* = 0.90).

The MOLLI in-vivo variation (53.7 ± 8.1 ms) shows no significant difference (*p* = 0.057) compared with SAPPHIRE (60.1 ± 8.7 ms), but a significant reduction compared with SASHA (70.0 ± 9.3 ms, *p* < <0.001). SAPPHIRE yields lower variation than SASHA (14 ± 10 %, *p* < 0.002). Both SR T_1_-mapping methods show a trend of increased variation in the inferior, inferior-lateral and anterior segments, compared with the septal and anterior-lateral segments.

Figure 3 (bottom row) shows the segmental ECV based on the first post-contrast session. MOLLI yields the lowest ECV-values, followed by SAPPHIRE and SASHA, with all differences being significant (*p* < 0.007). There was no significant difference between the inter-subject variability of the ECV-values obtained with MOLLI (0.026), SAPPHIRE (0.020) and SASHA (0.025) (*p* = 0.53).

A summary of T_1_-times and ECV-values for the native myocardium and both post-contrast times are given in Table 1. The second post-contrast imaging time showed a trend of higher ECV-values than the first time point, with an absolute deviation of 0.014 ± 0.016 for MOLLI (*p* < 0.001), 0.008 ± 0.013 for SAPPHIRE (*p* < 0.02), and 0.005 ± 0.020 for SASHA (*p* = 0.24).

**Table 1:** Myocardial and blood T_1_-times measured with MOLLI and two saturation-recovery techniques at 3T

			MOLLI	SAPPHIRE	SASHA
Native	T_1_-time [ms]	Myo	1182.6 ± 35.8	1578.1 ± 35.9	1522.8 ± 40.5
		Blood	1781.4 ± 135.7	2047.6 ± 132.0	1919.3 ± 134.2
Post 1 (~15 min)	T_1_-time [ms]	Myo	541.1 ± 33.8	746.2 ± 49.3	722.0 ± 57.2
		Blood	349.1 ± 34.4	387.4 ± 37.4	390.6 ± 42.6
	ECV [%]		26.0 ± 2.6	20.2 ± 2.0	21.3 ± 2.5
	GBCA Concentration [μmol/L]	Myo	288.5 ± 38.5	203.2 ± 27.9	210.2 ± 34.7
		Blood	665.4 ± 85.7	604.2 ± 72.6	590.4 ± 84.1
Post 2 (~25 min)	T_1_-time [ms]	Myo	581.5 ± 33.0	794.1 ± 46.9	773.2 ± 55.6
		Blood	405.6 ± 39.5	439.3 ± 39.8	441.7 ± 43.8
	ECV [%]		27.5 ± 3.1	21.0 ± 2.8	21.9 ± 3.0
	GBCA Concentration [μmol/L]	Myo	251.4 ± 38.5	179.7 ± 24.3	183.5 ± 31.0
		Blood	550.3 ± 75.5	515.9 ± 62.7	504.2 ± 72.4

The results from the inter- and intra-observer analysis are given in Table 2. High reproducibility in terms of agreement was shown for all three sequences, with mean differences <15 ms for native and <6 ms for post-contrast T_1_-mapping. Good intra-observer consistency was obtained in all scans (ICC > 0.97). Inter-observer consistency was slightly lower across the three sequences, especially for native T_1_-times (ICC > 0.94). No statistically significant difference was found among the three sequences, neither in terms of agreement nor consistency.

**Table 2:** Inter- and Intra-observer variability. The upper part of the table lists the results from the agreement analysis, based on absolute differences between the ROI sets. The lower part of the table depicts the consistency analysis, based on an ICC (Winer’s adjustment for anchor points). No significant difference was found among the sequences

		Geometric Mean of Absolute Difference [CI = 95 %]			*p*-value*
		MOLLI	SAPPHIRE	SASHA	
Inter	Native	11.3 [3.7–30.5]	13.0 [5.7–30.4]	8.8 [1.6–27.0]	0.27
	Post	2.8 [0.2–22.8]	5.3 [0.7–23.2]	5.3 [0.6–20.1]	0.19
Intra	Native	7.1 [1.2–18.7]	5.1 [0.7–16.4]	3.3 [0.3–13.3]	0.09
	Post	3.6 [1.2–13.5]	4.1 [0.5–18.8]	3.2 [0.5–17.1]	0.69
		ICC [CI = 95 %]			*p*-value**
		MOLLI	SAPPHIRE	SASHA	
Inter	Native	0.941 [0.858–0.976]	0.973 [0.932–0.989]	0.958 [0.898–0.983]	>0.10
	Post	0.983 [0.957–0.993]	0.968 [0.920–0.987]	0.986 [0.965–0.994]	> 0.07
Intra	Native	0.970 [0.926–0.988]	0.978 [0.945–0.991]	0.984 [0.960–0.994]	> 0.17
	Post	0.991 [0.977–0.996]	0.990 [0.975–0.996]	0.991 [0.978–0.997]	> 0.60

*One-way ANOVA on log of absolute difference, Significance level *p* < 0.05

**2-sided F-Statistics with Bonferroni correction, minimal *p*-value of three pair-wise tests is listed, Significance level *p* < 0.017

**Fig. 4:**
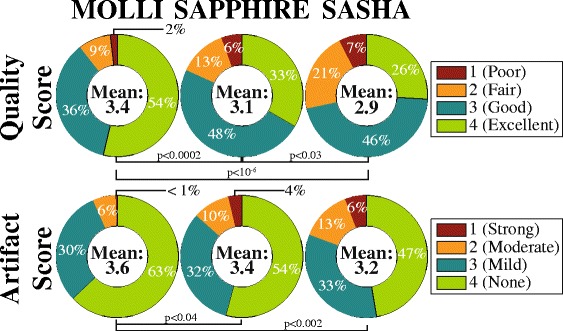
Pie charts showing the distribution and average of the quality (*top row*) and artifact (*bottom row*) scoring across all T_1_-maps and across both readers. Eighty-one percent of all images were scored with at least “good” image quality, with MOLLI having the highest average score and the lowest artifact scoring. SAPPHIRE shows higher average quality and similar artifact scores compared with SASHA

Figure 4 depicts the readers’ quality and artifact scores. All three sequences were scored with “good” image quality on the average. MOLLI resulted in the highest average quality scores, followed by SAPPHIRE. The SASHA method showed the lowest quality in visual assessment. All pair-wise differences were found to be significant (*p* < 0.03). The average artifact scoring was significantly better for MOLLI (3.6 ± 0.3) compared with SAPPHIRE (3.4 ± 0.3, *p* = 0.03) and SASHA (3.2 ± 0.3, *p* = 0.001). Example images illustrating the effect of off-resonance artifacts on the T_1_-maps are given in Additional file 3: Figure S3.

MoCo was successfully performed on almost all MOLLI imaging series (97 %) and on the majority of the SAPPHIRE data (82 %). However, only few SASHA imaging series were correctly registered using MoCo (8 %).

## Discussion

In this study, we assessed reference values and in-vivo precision of SR T_1_-mapping at 3T in comparison with MOLLI. SR T_1_-mapping provided robust image quality throughout the study. MOLLI T_1_-maps were shown to consistently provide the highest image quality rating and lowest artifact incidence. However, significantly better ex-vivo accuracy was confirmed for SR methods for the trade-off against a slight reduction of in-vivo precision. No significant difference was found in the inter-subject variability and the inter-and intra-observer variability among the three methods.

Native T_1_-times of the human myocardium using SR T_1_-mapping were found to be around 1550 ms. This reveals a field strength dispersion of approximately 30 % compared with 1.5T (1210–1220 ms [9]), which is in good agreement with reported literature values for cardiac tissue of animals [23, 24]. MOLLI T_1_-times from our own findings and previous reports at 3T (1166 ms [14]) demonstrate a significant underestimation of about 20–30 % compared with the present results of SR T_1_-mapping. This underestimation, as confirmed by the phantom study, indicates decreased in-vivo accuracy of MOLLI. SASHA T_1_-mapping was previously reported to have about 150 % higher in-vivo variability than MOLLI at 1.5T [10]. Our results demonstrate that the loss in precision when using SR over MOLLI is drastically reduced compared with 1.5T. The present results indicate that at 3T, MOLLI remains to provide higher visual image quality than SR methods. However, the high ex-vivo accuracy, the low level of precision-loss, and the good inter-subject variability, indicate only a small gap to SR T_1_-mapping. Hence, SR methods at 3T provide a valuable option for trading-off increased quantification accuracy against a reduction of overall image-quality.

Alternative T_1_-map reconstructions have been proposed for SR T_1_-mapping, to improve precision albeit at the cost of reduced accuracy and increased sensitivity of the T_1_-time to the choice of scan parameters. A two-parameter fit for SASHA T_1_-mapping was recently proposed [10] and initial results on an extension using a variable flip-angle scheme for the bSSFP imaging readout to minimize the loss in accuracy were presented [25]. Two parameter fitting has also been used for SAPPHIRE post-contrast T_1_-mapping [6]. However, imperfect inversion efficiency might impair the accuracy of SAPPHIRE, when using a two-parameter fit for native T_1_-mapping. The use of a predetermined correction factor for incomplete inversion, as previously proposed [17], might be warranted for this application.

The reported reference ECV values for MOLLI (~0.26) and SR T_1_-mapping (~0.21) obtained in this study are in good agreement with previous literature. For MOLLI, ECV-values between 0.25 and 0.27 have been reported at 1.5T [9, 15, 26] and between 0.26 and 0.28 at 3T [26–29]. Furthermore, the slight increase in ECV-values between the two post-contrast time points has been previously observed with MOLLI at 3T [29], and the ECV deviation between the two time points is in agreement with previous reports (0.258–0.272 for times between 10 and 25 min [28]. Close agreement of SAPPHIRE ECV-values are obtained with a previous study at 1.5T (ECV: 0.20 [9]). SASHA ECV-values were reported as 0.18 [9], 0.21 [30] and 0.22 [31, 32] in healthy subjects at 1.5T. The close agreement of these values with our results, as well as with ECV-values obtained with SR T_1_-mapping in an animal study at 3T (AIR: 0.20–0.21 [33]) proves high cross field-strength consistency for SR based ECV-measures.

Despite the higher precision of MOLLI compared to SR T_1_-mapping, previous studies did not report significant differences in the scan-rescan reproducibility [9, 26]. To add on this, our results show no significant difference in the inter- or intra-observer variability between the methods either. All three methods showed consistency with ICCs > 0.90, which is considered excellent for diagnostic tools [34]. The values of observer variability characteristics obtained in this study are well in line with previous reports [27, 35–38]. However, some studies from specialized centers achieved consistently higher inter- and intra-observer variability, with ICCs > 0.99 [27, 29, 39]. This difference might be explained by the limited clinical experience of our readers. Therefore, extensive observer training and an extensive common learning phase for both readers seems to be required to achieve optimal reproducibility results in T_1_-mapping.

Imaging at 3T using bSSFP has considerable challenges compared with 1.5T. Off-resonance artifacts are commonly induced by magnetic susceptibilities at tissue interfaces, e.g. epicardium-lung interface. In this study, frequency scouts were used to minimize off-resonance artifacts. However, careful volumetric shimming is still essential at 3T to ensure robust image quality. Also, the rapid imaging readout reaches specific absorption rate (SAR) limitations at 3T. As SR T_1_-mapping methods were shown to be independent of the imaging flip-angle [12], improved imaging SNR could potentially be achieved using optimized excitation pulses with higher flip-angles and low SAR, for the trade-off against suboptimal slice profiles.

Non-rigid motion correction algorithms, as used in this study, are dependent on strong contrast within the area of interest [40]. Hence, MoCo was more effective for MOLLI than for the SR methods. Tailored motion correction algorithms might be required if a further reduction of residual motion in the SR imaging series is necessary.

This study has several limitations. Due to the lack of feasible methods for the assessment of “true” T_1_-times in the myocardium, no direct evidence of the in-vivo accuracy of SR methods can be given. Instead, phantom accuracy was used as an indicator of in-vivo accuracy. Evaluation of the sequence characteristics was restricted to accuracy and precision, specifically no inter- or intra-session reproducibility was considered in this study. A tightly controlled cohort of young healthy volunteers was recruited for the study, in order to obtain reproducible reference values of the healthy myocardium that are not affected by potential age-related fibrosis in the muscle. As the T_1_-time of the myocardium is known to be age and sex dependent [41], cohorts that are age/sex matched to the particular patient population are to be assessed if more specific T_1_-reference values with reduced intra-cohort variability are required.

## Conclusions

Saturation-recovery at 3T was shown to provide accurate and robust T_1_-map quality at a field-strength of 3T. In-vivo comparison to MOLLI showed decreased subjective image quality scores, a slight loss in precision, but comparable inter-subject, inter-, and intra-observer variability.

## Additional files

Additional file 1: Phantom Study. Methods and results of the phantom study examining saturation efficacy of the saturation modules and accuracy and precision of SAPPHIRE, SASHA and MOLLI in comparison to spin-echo reference scans. (DOCX 102 kb)

Additional file 2: Scoring Criteria. Detailed definition of criteria for T_1_-map quality and artifact scoring. (DOCX 25 kb)

Additional file 3: Susceptibility Artifacts. Images showing the influence of frequency shifts and susceptibility artifacts on baseline images and T_1_-maps. (DOCX 113 kb)

## Abbreviations

AHA: American Heart Association
ANOVA: Analysis of variance
bSSFP: Balanced Steady-State Free Precession
CMR: Cardiovascular magnetic resonance
ECV: Extracellular volume fraction
GBCA: Gadolinium based contrast agent
GRAPPA: Generalized Autocalibrating Partially Parallel Acquisition
ICC: Intraclass correlation coefficient (ICC)
IR: Inversion-recovery
MoCo: Motion Correction
MOLLI: Modified Look-Locker inversion recovery
SAPPHIRE: Saturation-Pulse Prepared Heart-rate independent Inversion-Recovery
SAR: Specific absorption rate
SASHA: Saturation-recovery Single-SHot Acquisition
ShMOLLI: Shortened MOLLI
SNR: Signal-to-Noise ratio
SR: Saturation-recovery
WET: Water suppression Enhanced through T_1_-effects
