# Supplementary material for: Myocardial T1-mapping at 3T using saturation-recovery: reference values, precision and comparison with MOLLI
Source: J Cardiovasc Magn Reson. 2016 Nov 18;18:84. doi: 10.1186/s12968-016-0302-x (PMC5114738; doi:10.1186/s12968-016-0302-x)
Supplement: Supplementary file 1 — Phantom Study. Methods and results of the phantom study examining saturation efficacy of the saturation modules and accuracy and precision of SAPPHIRE, SASHA and MOLLI in comparison to spin-echo reference scans. (DOCX 102 kb) [file 12968_2016_302_MOESM1_ESM.docx]

# Additional file 1 – Phantom Study

## Methods

#### Phantom imaging was performed in nine test vials containing Gd-doped agarose gel of various concentrations. Reference T_1_-values were obtained from an inversion-recovery spin-echo series (TE/TR/α=15ms/10s/90°; TI=30,50,100,200,...,6400ms,None) using a three-parameter fit. Reference T_2_-values were acquired using a Carr-Purcell-Meiboom-Gill (CPMG)-sequence with 32 echoes (TR/α=10s/90°; TE=25,37.5,50,62.5,...,400ms). The first echo time (TE=12.5ms) was excluded from evaluation, due to stimulated echoes.

#### Saturation Efficacy

Three saturation modules were studied for saturation-recovery T_1_-mapping at 3T: 1) Two composite WET pulses comprised of 4 RF pulses with interleaved spoiling 2) one adiabatic plane-rotation (BIR4) 90° pulse. The WET pulses applied the following flip-angles: 72°, 92°, 126°, 193° (“WET4a”, ([1](#_ENREF_1))), 120°, 90°, 180°, 230° (“WET4b”,([2](#_ENREF_2))). The relative SAR was 1.0, 1.6 and 8.8 for the WET pulses and the BIR4, respectively. The saturation efficacy was calculated from a three-point saturation-recovery fit based on the reference T_1_-time and the image intensities of two spin-echo sequences, one with and one without saturation preparation (TE/TR=15ms/10s, TS=6ms≙duration of post-spoiler).

#### Accuracy and Precision

T_1_-maps for the assessment of phantom accuracy and precision were acquired using SAPPHIRE, SASHA and MOLLI with the same imaging parameters as for the in-vivo study. 20 repetitions of each sequence were performed with a simulated ECG signal at 60 bpm. Relative accuracy was defined as the relative difference between the spin-echo reference value and the mean T_1_-time within a manually drawn ROI averaged over all repetitions. Relative precision was defined as the standard-deviation of the mean T_1_-time per vial across the repetitions divided by the reference T_1_. Accuracy and precision between the three methods were statistically compared using an ANOVA and a Kruskal-Wallis test, respectively. For a p-value <0.05 in the group test, pair-wise Student’s t-Test and Wilcoxon-Signed-Rank test were performed at a significance level of 0.05.

## Results

#### Saturation Efficacy

Table S1 summarizes the saturation efficacy of the four saturation modules. Good signal suppression with less than 3% residual signal after saturation was observed with all pulses over a wide range of T_1_-times. The WET4a pulse showed the best signal suppression with the lowest SAR burden.

Table S1: Saturation efficacy of four saturation modules in spin-echo measurements of 9 phantom vials. All four modules result in thorough signal suppression (η > 0.99), with residual signal of at most 2.4 %.

| **T1 Time** | **Saturation Efficacy** | | |
| --- | --- | --- | --- |
|  | **WET4a** | **WET4b** | **BIR4** |
| 114 | 1.000 | 0.995 | 0.999 |
| 207 | 0.999 | 0.994 | 0.997 |
| 383 | 0.995 | 0.999 | 0.987 |
| 692 | 1.000 | 0.999 | 0.997 |
| 1287 | 1.000 | 0.993 | 0.995 |
| 1863 | 0.995 | 0.987 | 0.990 |
| 2327 | 0.992 | 0.985 | 0.997 |
| 2765 | 0.989 | 0.985 | 0.996 |
| 3188 | 0.991 | 0.989 | 0.989 |
| **Mean±Std** | **0.996±0.004** | **0.992±0.006** | **0.994±0.004** |

#### Accuracy and Precision

Figure S1 depicts accuracy and precision of the MOLLI, SAPPHIRE and SASHA sequences. MOLLI shows underestimation up to 321ms (10%). Good accuracy was observed with the saturation-recovery sequences (deviation: SAPPHIRE<1.9% and SASHA<3.9%).

MOLLI shows the lowest T1-time variation: 29% lower than with SAPPHIRE (p=0.090) and 50% lower than with SASHA (p<0.024). SAPPHIRE yielded slightly better precision than SASHA (30% lower variation).


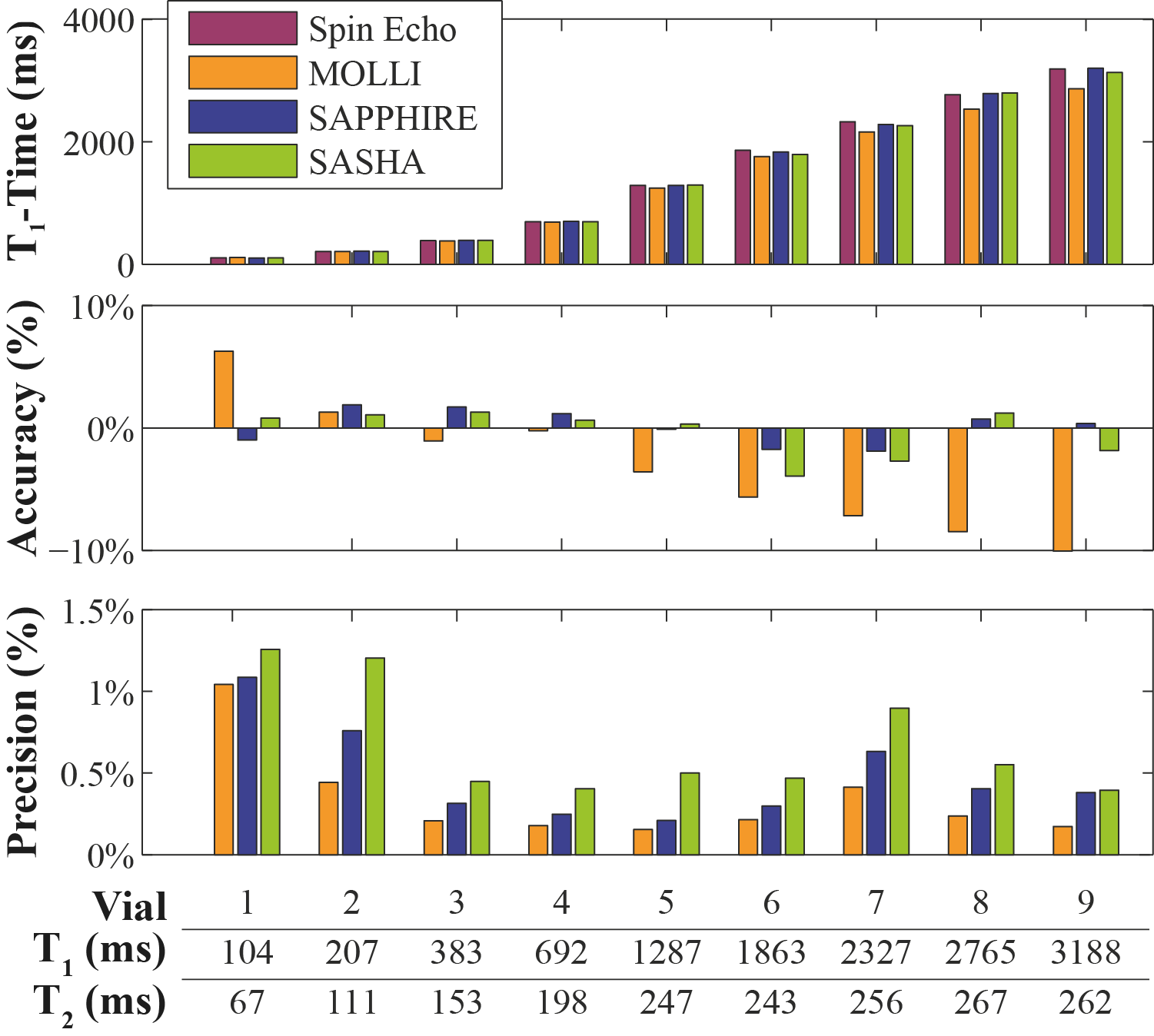


Figure S1: T_1_-times, relative accuracy and relative precision in comparison to reference spin-echo scans in nine phantom vials. The reference T_1_- and T_2_-times are given in the bottom. MOLLI deviates from the spin-echo reference by up to 10%. Improved accuracy was obtained using the saturation-recovery methods (<3.9%). MOLLI shows the best precision. SAPPHIRE results in lower T_1_-time variation than SASHA.

## References

1. Weingärtner S, Akcakaya M, Basha T, et al. Combined saturation/inversion recovery sequences for improved evaluation of scar and diffuse fibrosis in patients with arrhythmia or heart rate variability. Magn Reson Med. 2014;71(3):1024-1034.

2. Sung K, Nayak KS. Design and use of tailored hard-pulse trains for uniformed saturation of myocardium at 3 Tesla. Magn Reson Med. 2008;60(4):997-1002.
