# Supplementary material for: Myocardial T1-mapping at 3T using saturation-recovery: reference values, precision and comparison with MOLLI
Source: J Cardiovasc Magn Reson. 2016 Nov 18;18:84. doi: 10.1186/s12968-016-0302-x (PMC5114738; doi:10.1186/s12968-016-0302-x)
Supplement: Supplementary file 2 — Scoring Criteria. Detailed definition of criteria for T1-map quality and artifact scoring. (DOCX 25 kb) [file 12968_2016_302_MOESM2_ESM.docx]

# Additional file 2 – Scoring Criteria

## Methods

Quality scores were given based on the following criteria, which are adapted from ([1](#_ENREF_1)):

- **“4 (excellent)** is assigned when the myocardial borders are crisp and distinct from the blood pool with less than or equal to 1 pixel of partial volume border and no noticeable geometric distortion. The myocardial signal has to be homogeneous in areas assessed to be normal tissue.”
- **“3 (good)** is assigned when one of the above criteria is absent, i.e., there are 1–2 pixels of representative myocardial T_1_ with slightly increased border between blood and myocardium, or geometric distortion is present but not impacting the T_1_ values, or there are small regions (10%) of inhomogeneity in the myocardium.”
- **“2 (fair)** means that that 2 criteria under the excellent category are absent, i.e., only a thin stripe of myocardium is present from which to sample the representative T_1_, or geometric distortion is present, or multiple regions have inhomogeneity deemed to be artifactual but affect less than 50% of the myocardium.”
- **“1 (poor)** means that inhomogeneities or geometric distortion affect greater than 50% of the myocardium making it difficult to distinguish regions of normal myocardium from noise or artifacts.”

To score the susceptibility artifact severity, the readers were provided with the following criteria:

- **1 (strong)**: Transmural artifacts prevent reliable evaluation in a region greater than 15% of the myocardium.
- **2 (moderate)**: Transmural artifacts hamper regional evaluation of the myocardium, in a significant area, but < 15%.
- **3 (mild):** Mild artifacts corrupt small parts of the myocardial thickness or over a small volume.
- **4 (none):** No susceptibility artifacts affect the myocardium.

## References

1. Kellman P, Wilson JR, Xue H, Ugander M, Arai AE. Extracellular volume fraction mapping in the myocardium, part 1: evaluation of an automated method. J Cardiov Magn Reson. 2012;14:63.
