# Supplementary material for: Myocardial T1-mapping at 3T using saturation-recovery: reference values, precision and comparison with MOLLI
Source: J Cardiovasc Magn Reson. 2016 Nov 18;18:84. doi: 10.1186/s12968-016-0302-x (PMC5114738; doi:10.1186/s12968-016-0302-x)
Supplement: Supplementary file 3 — Susceptibility Artifacts. Images showing the influence of frequency shifts and susceptibility artifacts on baseline images and T1-maps. (DOCX 113 kb) [file 12968_2016_302_MOESM3_ESM.docx]

# Additional file 3 – Susceptibility Artifacts


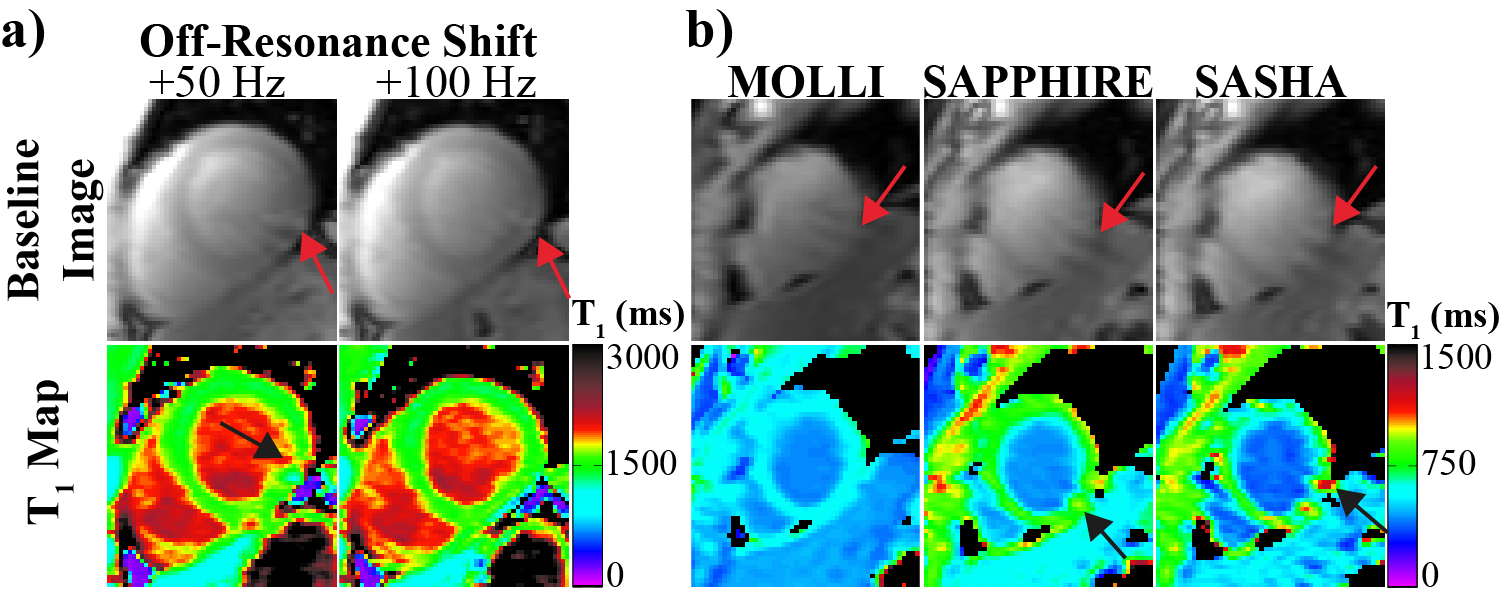


Figure S3: a) Images showing the effect of two different frequency shifts on baseline images (top row) and on the resulting native SASHA T_1_-maps (bottom row). Susceptibility artifacts are readily visible at +50 Hz (red arrow), leading to destructive artifacts in the corresponding T_1_-map (black arrow). No such artifact is visually apparent in the T_1_-map at +100 Hz, due to reduced artifact intensity in the baseline image (red arrow).

b) Images showing the influence of susceptibility artifacts on the baseline images (top row) and on the resulting post-contrast T_1_-maps (bottom row) for all three T_1_-mapping sequences. The susceptibility artifacts are of visually comparable severity in the baseline images for all three sequences (red arrows). However, the artifact has almost no effect on the MOLLI T_1_-map. Mild artifacts are observed in the SAPPHIRE T_1_-map and strong artifacts in the SASHA T_1_-map, as indicated by the black arrows.
